# Man vs. machine: can AI outperform ESL student translations?

**DOI:** 10.3389/frai.2025.1624754

**Published:** 2025-07-09

**Authors:** Anas Alkhofi

**Affiliations:** Department of English, College of Arts, King Faisal University, Al-Ahsa, Saudi Arabia

**Keywords:** AI in language education, machine translation, Google Translate, translation pedagogy, instructor perceptions, English-to-Arabic AI translation

## Abstract

This study compares the quality of English-to-Arabic translations produced by Google Translate (GT) with those generated by student translators. Despite advancements in neural machine translation technology, educators often remain skeptical about the reliability of AI tools like GT and often discourage their use. To investigate this perception, 20 Saudi university students majoring in English and Translation produced human translations in Arabic. These student-generated translations, along with their GT equivalents, were rated by 22 professors with experience in language-related fields. The analysis revealed a significant preference for GT translations over those produced by students, suggesting that GT’s quality may exceed that of student translators. Interestingly, while GT translations were consistently rated higher, instructors often misattributed the better translations to students and the poorer ones to GT. This reveals a strong perceptual bias against AI-generated translations. The findings support the inclusion of AI-assisted translation tools in translation training. Incorporating these tools will help students prepare for a job market where AI is playing an increasingly important role. At the same time, educators should adopt strategies incorporating AI tools without sacrificing the development of students’ core translation skills.

## Introduction

Artificial intelligence (AI) has become integral to our everyday lives. It affects various activities, from personal tasks to professional endeavors. In educational settings, the use of AI has been particularly transformative. AI enhances learning experiences and introduces new tools for both educators and students. One area where AI has seen significant advancement is in the field of machine translation. Neural machine translation, a widely used AI-based method, employs neural networks to learn the complex statistical relationships between words in different languages. As a result, current AI-based translation tools can produce translations that are not only more accurate but also more naturally sounding when compared to older machine translation methods.

Google Translate (GT) is one of the most widely used AI-based translation tools (Lee and Briggs, 2021). In 2016, it transitioned from statistical machine translation to neural machine translation. This shift has allowed GT to produce more accurate and fluent translations. Despite this improvement, many educators hold low expectations for GT and often advise students to avoid it (Ducar and Schocket, 2018; Kaspere and Liubiniene, 2023; Lee and Briggs, 2021). Nevertheless, students frequently rely on such tools, even when instructors explicitly discourage their use (Correa, 2014; Kazemzadeh and Fard Kashani, 2014; Niño, 2009; Salinas and Burbat, 2023).

While GT has been widely explored as a pedagogical tool, (Bahri and Mahadi, 2016; Cancino and Panes, 2021; Lee, 2020; Stapleton and Kin, 2019; Ting and Tan, 2021; Tsai, 2019; Van Lieshout and Cardoso, 2022) relatively less attention appears to have been given to its use among students majoring in translation (Mulyani and Afina, 2021; Man et al., 2020). In addition, there seems to be limited research directly comparing the quality of machine translations, such as those produced by GT, with translations produced by student translators.

Given that AI-based translation quality varies across language pairs (Mezeg, 2023; Wang et al., 2022), this study focuses on English-Arabic, a translation pair that has yet to be fully explored. Specifically, this research seeks to explore how translation instructors evaluate different translations, some of which—unknown to the instructors—have been produced by GT, while others have been generated by translation students. The following research questions guide this study: 1. How do instructors rate the quality of English-to-Arabic translations produced by Google Translate compared to those produced by students? 2. Is the instructors’ perception of translation origin (student vs. machine) associated with their ratings?

## Literature review

### Google translate in language education

Several studies have emphasized the positive impact of GT on vocabulary learning and writing proficiency. For instance, Van Lieshout and Cardoso (2022) demonstrated that GT, when used alongside its text-to-speech and speech recognition features, could support self-directed learning of Dutch vocabulary and pronunciation. In the study, students were given 10 target phrases and used GT to listen to the pronunciation, repeat the phrases aloud, and receive automated feedback through speech recognition. This cycle of listening, speaking, and checking helped students engage actively with the new language, resulting in significant vocabulary gains immediately after the session and moderate retention after 2 weeks. Similarly, Ting and Tan (2021) investigated the use of GT among indigenous learners in Malaysia and found that it significantly improved English vocabulary acquisition. Their findings emphasize GT’s potential to support learner-centered approaches, especially in contexts with limited access to traditional learning resources.

In the context of writing, several studies have reported that GT can enhance the quality of student texts, particularly in terms of vocabulary and grammatical accuracy. Lee (2020) investigated how Korean-English college students used GT to improve their L2 writing. Students first translated their L1 texts into English without GT, then used GT to produce a second version. By comparing and revising their work against the GT output, they reduced lexicogrammatical errors and improved their writing. The study also noted that GT encouraged students to view writing as a process, facilitating more thoughtful revisions. Similarly, Tsai (2019) reported that Chinese EFL students produced higher-quality texts with GT, particularly in vocabulary richness and grammatical accuracy. Supporting this trend, Stapleton and Kin (2019) examined the use of GT among Chinese primary school EFL learners. One group wrote directly in English, while the other wrote in Chinese and used GT to translate into English. Teachers, unaware of the texts’ origins, rated both versions similarly. In cases where ratings differed, GT texts often scored higher. These results suggest that GT texts can match, and sometimes surpass, the quality of student writings.

Despite these positive findings, the literature also points to several challenges and limitations associated with using GT in language learning. Bahri and Mahadi (2016) cautioned against over-reliance on GT, particularly among beginners learning Malay. Although GT helped develop written skills, it proved less effective for supporting grammar and oral language development. Similarly, Cancino and Panes (2021) found that while GT can enhance syntactic complexity and accuracy in EFL writing, its benefits depend heavily on proper instruction. Without sufficient guidance, learners may struggle to use the tool effectively, reinforcing the need for active teacher involvement when incorporating AI-based tools into the classroom.

Concerns about the effect of GT on learner motivation and the development of core language skills are common in the literature. Stapleton and Kin (2019) pointed out that relying heavily on GT might lower students’ motivation to learn how to write in the target language. This issue is especially important in light of Lee’s (2020) findings, which suggested that although GT can support some aspects of language learning, it may also lead to a shallow understanding of language structures if used without proper guidance.

### Previous research comparing machine translation with professional human translation

Research on machine translation quality has produced mixed results, often influenced by the timing of the studies. Early research emphasized poor output quality, citing issues like literal translations, structural problems, and inappropriate word choices (Kliffer, 2005, 2008; Niño, 2008, 2009). However, the introduction of neural machine translation in 2016 marked a turning point, significantly improving accuracy. Using machine learning and large text corpora, current machine translation systems are better at considering context, reducing the frequency of literal translations (Ducar and Schocket, 2018; Koehn et al., 2020). Accordingly, this review focuses on studies published after 2016.

Several studies have compared the output of machine translation systems with human translations. For example, Hassan et al. (2018) found that Microsoft’s system produced Chinese-to-English news translations at a quality level comparable to professional human translators. Läubli et al. (2018) supported this finding, noting strong sentence-level performance. However, they emphasized that human translators still outperform machines at the document level, particularly in maintaining coherence and contextual flow.

Similarly, Graham et al. (2020) found that top-performing machine translation systems are approaching human parity, especially in language pairs such as English-Russian and English-German. Their analysis showed that although minor random errors remain, there were no consistent patterns distinguishing human and machine translations in these languages, highlighting the robustness of machine translation. However, these studies show that the effectiveness of machine translation can vary significantly across language pairs. While systems perform well with widely studied pairs like English-Russian and English-German, they struggle with less commonly studied languages. In media and entertainment, Calvo-Ferrer (2023) demonstrated that viewers were often unable to distinguish between subtitles generated by machine translation and those produced by professional translators, particularly in translations of humor and cultural references.

### Previous research comparing machine translation with student translations

In an educational context, Lee and Briggs (2021) compared machine translation outputs with student translations from Korean to English and found that machine translation often outperformed intermediate learners in mechanics, vocabulary, and grammar. This suggests that machine translation could serve as a high-quality model for students to learn from. Similarly, Borodina et al. (2021) conducted an experimental study that shows the potential of GT to improve translation training. In this study, students were divided into two groups: an experimental group that used GT and a control group that did not. The results showed that the English-Russian translations produced by students in the experimental group were better in all respects than those produced by the control group. Using GT helped reduce errors and improve the accuracy of industry-specific terminology. That said, Alsalem (2019) warned that students who rely heavily on GT for initial drafts may overlook essential translation skills, such as using dictionaries or evaluating word choices critically. These skills are important for building professional competence in translation.

#### The role of Post-editing in machine translation

Given the widespread use of machine translation tools like GT among students, post-editing has become a practical and necessary skill. Rather than banning these tools, educators can guide students to engage critically with machine translation output by identifying and correcting errors. This approach strikes a balance between leveraging machine translation’s benefits and preserving core translation competencies’ development. Jia et al. (2019) compared post-editing of machine translation output with translation from scratch and found that post-edited translations achieved equivalent fluency and accuracy. Their study also showed that students generally had a positive attitude toward post-editing, and that the quality of post-edited translations was comparable to those completed entirely by human translators. The researchers strongly advocate for the inclusion of post-editing training in university curricula, suggesting that systematic instruction can help students save time and reduce cognitive effort compared to translating from scratch. Moreover, Jia et al. (2019) research aligns with Yamada’s (2019) findings, as both studies highlight the need for translation training that develops the specific skills required for effective post-editing.

Khoury et al. (2024) highlight the importance of hands-on post-editing experience, especially for specialized texts like legal translations. Their findings suggest that while general translation skills are useful, focused training in post-editing is essential to produce high-quality translations in specialized fields. This is consistent with Salinas and Burbat (2023), who argue that despite past skepticism toward machine translation, its role in translation education deserves renewed attention. They suggest that post-editing machine translation output can be a valuable teaching tool, helping students build critical thinking and language skills. Salinas and Burbat (2023) also note that while machine translation cannot replace the creativity and cultural sensitivity of human translators, it can be effective when paired with post-editing. That said, the human-like errors generated by current machine translation systems can be particularly challenging for students to identify and correct, which underscores the need for thorough training in post-editing to meet professional standards (Yamada, 2019).

### Challenges and limitations of machine translation

Despite recent advancements, the literature highlights several challenges and limitations of machine translation systems. Mezeg (2023) found that although tools like Google Translate and DeepL have improved, they still struggle with complex language pairs such as French-Slovene, particularly in terms of lexical choice and stylistic accuracy. This underscores the ongoing need for post-editing and suggests that translation education should increasingly equip students with post-editing skills, as this is likely to become a central task for future translators.

Similarly, Labarta Postigo (2022) emphasizes machine translation limitations when handling specialized language, such as metaphors and idiomatic expressions. The study showed that machine translation systems continue to face difficulties with these nuanced elements, which often require the deep cultural and contextual understanding that only human translators can provide. This finding aligns with Sabtan et al. (2021), who evaluated machine translation performance in translating colloquial Arabic to English and identified significant issues, including inaccurate equivalents, unnecessary additions, and transliterations. These errors reflect the ongoing challenges machine translation systems face when processing language varieties that deviate from standard forms.

A recurring theme across the literature is the variability in machine translation quality depending on the language pairs involved. While studies such as those by Hassan et al. (2018) and Graham et al. (2020) highlighted the high quality of machine translation for language pairs like English-Russian and English-German, other studies revealed significant challenges when translating between more complex or less common language pairs, such as French-Slovene (Mezeg, 2023) and Arabic-English for colloquial varieties (Sabtan et al., 2021). Alsalem (2019) also reported that GT’s Arabic-English translations are far from perfect, emphasizing the difficulties machine translation systems face when processing languages with complex grammatical structures and morphological variations like Arabic. This suggests that while machine translation has made significant progress, its effectiveness is not uniform across all language pairs.

Building on these observations, it is clear that certain research areas remain insufficiently explored. While previous studies have examined the use of GT in language learning and its comparison with professional translations, relatively little attention has been given to direct comparisons between GT outputs and student translations, particularly in the English-to-Arabic language pair. The current study investigates how instructors evaluate the quality of English-to-Arabic translations produced by GT versus student translations. Additionally, since, to our knowledge, no studies have specifically examined the relationship between instructors’ perceptions of translation origin and their actual evaluation of translation quality, the present study explores whether perception is associated with rating outcomes.

## Methods

### Participants

The student translations were produced by 20 students (16 males and four females) selected through convenience sampling. These students were enrolled in a final translation project course, and the researcher collected the available translations completed for a previous class assignment. In this project, each student translated at least two book chapters from English into Arabic. All participants were Saudi university students majoring in English and Translation at the bachelor’s level. Although the students had varying levels of proficiency, their enrollment in the final course indicates that they had completed the necessary introductory courses in translation and related subjects, suggesting a solid foundation in both languages.

Additionally, 22 instructors (12 females and 8 males) from the same department were recruited to evaluate the translations. Among them, 15 hold a Ph.D. and 5 hold a master’s degree, all in linguistics-related fields. Each instructor has a minimum of 6 years of teaching experience, ensuring that their evaluations were informed by substantial expertise in language and translation.

### Materials and procedures

The study materials consisted of 22 translation samples collected from 20 students. These samples were drawn from in-class translation assignments in which students translated full paragraphs into Arabic. For the purpose of this study, one or two unique sentences were extracted from each student’s work, resulting in a total of 22 sentences. These same sentences were then translated using GT, yielding a total of 44 translations—22 human-generated and 22 machine-generated. Each of the 22 professors evaluated all 44 translations, resulting in a total of 968 individual ratings, which were used for the statistical analysis.

To facilitate the evaluation process and minimize potential fatigue among the instructor participants, the translations were distributed across three separate Google Forms. Some instructors completed all three forms in one sitting, while others took up to 3 days, working at their own pace. The forms were sent electronically; after completing one form, each instructor received the next, until all 22 instructors had completed the three forms. To prevent order effects from influencing the evaluations, the sequence of the forms and the order of sentences within each form were randomized. An example of item presentation is shown below.

The English sentence:

Scott was 33 when he died 2 years ago. A long-time heroin user, he was forced to do without the drug for several weeks. He was killed by his first dose after being released from a five-week prison term.

First translation:

كانسكوت يبلغ من سنة 33 العمر عندما فارق الحياة قبل سنتين، متعاطٍ للهروين لمدة أجبر. طويلة على عدم استخدامها لمدة خمسة أسابيع خلال فترة قضاها في ولقد. السجن لقى حتفه بعد أول جرعة استخدمها بعد أن تم إطلاق صراحه.

Second translation:

سكوتكان يبلغ من عامًا 33 العمر عندما توفي قبل كان. عامين متعاطي الهيروين لفترة طويلة، واضطر إلى الاستغناء عن المخدر لعدة قُتل. أسابيع بجرعته الأولى بعد إطلاق سراحه من عقوبة السجن لمدة خمسة أسابيع.

After reading the second translation, instructors were presented with two separate rating scales, one for each translation. Each scale asked them to rate the translation quality on a scale from 1 (very bad) to 5 (very good).

After rating both translations, instructors were prompted to answer the question: “Which of the following is true?” The available options were: (1) “The first translation seems like it’s machine generated,” (2) “The second translation seems like it’s machine generated,” or (3) “Not sure.” This item was designed to assess whether instructors’ perceptions of translation origin were associated with their rating decisions.

## Results

The current study investigates how instructors rate the quality of translations produced by their students compared to GT translations. Table 1 presents the mean, median, and standard deviation for each group, showing that GT translations were rated more favorably by instructors.

**Table 1:** Descriptive statistics.

	Mean	Median	Std. Deviation	Minimum	Maximum
Student	2.769	3	1.225	1	5
Machine	3.728	4	1.128	1	5

**Figure 1:**
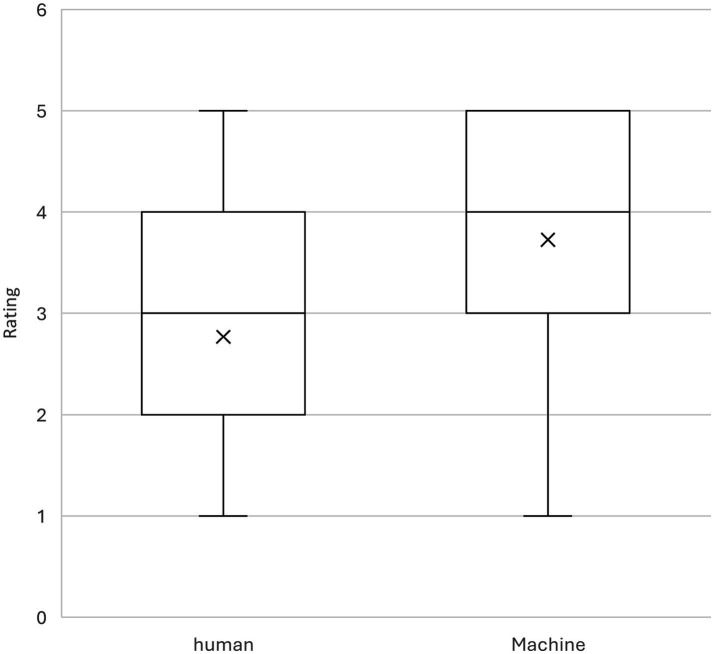
Boxplot of the ratings received for machine-generated translations vs human-generated translations.

Additionally, Figure 1 provides a visual representation of the spread and distribution of the ratings. The boxplot shows that ratings for machine translations were generally higher and less variable compared to student translations. The median rating for machine translations was 4, while the median for student translations was 3. The interquartile range was also slightly narrower for machine translations, suggesting more consistent evaluations.

Using the R statistical software, a linear mixed-effects model was employed to assess the differences in ratings between student and GT translations. In this model, translation type (student vs. machine) was treated as a fixed effect, while participants and sentences were treated as random effects to account for the repeated measures design. The dependent variable was the rating assigned to each translation. As shown in Table 2, the results indicate a statistically significant effect of translation type on the ratings, with machine translations rated significantly higher than student translations, t(893.13) = 13.41, *p* < 0.001. The effect size, Cohen’s d = 0.45, suggests a medium effect.

**Table 2:** Fixed effects estimates.

Term	Estimate	*SE*	*df*	*t*	*p*
(Intercept)	2.746	0.105	30.89	26.04	<001
Machine	0.959	0.072	893.13	13.41	<001

The study also investigated whether instructors’ perceptions of translation origin (machine vs. student) were associated with their ratings.

A linear mixed-effects model was then fitted to examine the relationship between perception and ratings. The results showed a significant association: translations perceived as student-generated received significantly higher ratings than those perceived as machine-generated, t(295.98) = 10.91, *p* < 0.001.

## Discussion

The first research question guiding this study asked: How do instructors rate the quality of English-to-Arabic translations produced by Google Translate compared to those produced by students? The results show that instructors preferred GT translations over those produced by their students. Both the mean and median ratings favored GT, and the significance testing confirmed this preference statistically. This outcome challenges the common doubts instructors might hold regarding the quality of GT. While this does not imply that GT’s quality is flawless, it does suggest, at the very least, that GT can outperform translations produced by students majoring in English and Translation, even at the final stages of their academic training.

The second research question asked: Is the instructors’ perception of translation origin (student vs. machine) associated with their ratings? The results of this study reveal an interesting disconnect between instructors’ perceptions and the actual quality of translations. Although the machine-generated translations received significantly higher ratings overall, instructors often attributed stronger translations to students and weaker ones to the machine. In other words, instructors often associated lower-quality translations with machine translation, even though these translations were, in fact, produced by students. Similarly, Alkhofi (2025) found that although the majority of instructors (90%) expressed confidence in their ability to detect student use of machine translation, their actual success rate did not exceed 25%.

This discrepancy highlights a critical issue in the evolving role of AI in educational and professional contexts: instructors’ perceptions of AI capabilities may not align with current technological advancements. As Alkhofi (2024) reported, nearly half of ESL instructors (45%) viewed GT as an ineffective tool for learning. Furthermore, the majority (73%) had never used class time to demonstrate how to use GT, and a significant portion (60%) had not assigned any tasks that involved using GT or similar online translation tools. Preconceived ideas about machine translation quality may reinforce educators’ reluctance to integrate it into teaching, clouding their judgment about its role in translation education. Vieira (2019) and Stapleton and Kin (2019) emphasized that AI-based tools are likely to become an integral part of modern translation practice. Therefore, the sooner instructors embrace these technologies and explore ways to incorporate them thoughtfully into their teaching, the better prepared students will be to succeed in a translation marketplace increasingly influenced by AI.

Recent studies align with the findings of the present research, showing that machine translation tools, such as GT, often outperform student translations or writing. For example, Lee (2020) compared the quality of GT translations from Korean to English with translations produced by intermediate-level ESL Korean students. The study found that while both sets of translations were equally comprehensible, GT consistently outperformed the students in several key areas, including mechanics, vocabulary, and grammar. Similarly, Stapleton and Kin (2019) conducted a study with primary school students, comparing GT Chinese to English translations with original English scripts written by the same students. The teachers, unaware that some scripts were GT-generated, did not differentiate between the two, often rating the GT translations higher. While these studies involved younger participants who may not yet have developed advanced writing skills in their mother tongue, the current study showed that such a trend of GT superiority persists even among older university students. Additionally, these results echo the broader conclusions indicated by Hassan et al. (2018), Barrault et al. (2019), and Graham et al. (2020), which show that machine translation performance is nearing the quality of professional human translations.

These findings challenge the traditional skepticism that many instructors hold toward machine translation. Historically, educators have regarded tools like GT with suspicion (Ducar and Schocket, 2018; Kaspere and Liubiniene, 2023; Lee and Briggs, 2021). However, the results of the current study suggest that these pedagogical attitudes need to be reconsidered. The quality of machine translation has evidently improved to a point where it can serve as a viable complement to human translation. Translation training should aim to strike a balance: educators must encourage the use of machine translation while also guarding against overreliance. At the same time, they must ensure that students remain competitive in an evolving marketplace where machine translation is becoming increasingly essential (Vieira, 2019). In line with Muftah’s (2022) recommendation, machine translation tools should be seen as supportive instruments within the translation process, with human translators taking on the role of post-editors. Machine-generated outputs can provide a useful starting point for professionals to refine the writing style and, importantly, adapt the content to fit the specific context and audience of the target language.

Growing evidence highlights the importance of incorporating post-editing into translation education as a balanced approach between relying entirely on machine translation and avoiding it altogether. Yang et al. (2023) found that post-editing machine translation significantly reduces translation learners’ processing time and mental workload, making it a preferred method over manual translation for translation students. This finding aligns with broader trends in professional translation workflows, where post-editing has become an integral part of the process (Koponen, 2016; Vieira, 2019). Drawing a parallel from another field, Stapleton and Kin (2019) observed that technology’s role in modern statistics courses shifted from manual calculations to understanding concepts through software tools like SPSS, leading to significant behavioral changes among students. In a similar way, the translation industry is evolving, with machine translation likely handling the initial drafting while human translators focus on refining and adapting the output. Given these developments, it is crucial for translation training programs to integrate machine translation tools and post-editing practices into their curricula, enabling students to benefit from these technologies rather than discouraging their use.

### The role of translation directionality in machine translation quality

Alsalem (2019) reported that Arabic-English translations produced by GT are far from perfect. However, Alsalem’s study focused on the translation of texts from Arabic into English, where the input to GT was Arabic. The complexity of Arabic grammar and morphology poses significant challenges for machine translation systems, mainly because Arabic datasets are less comprehensive compared to English ones. Zakraoui et al. (2020) support this point, noting that Arabic’s complex morphology, syntax, and lexical properties present substantial difficulties for machine translation systems. They emphasized that Arabic’s rich morphological structure and syntactic complexity, which differ greatly from English, often result in inadequate translation outputs when Arabic serves as the input language. Consequently, when translating from Arabic to English, GT and other machine translation systems often struggle to capture nuanced meanings and cultural contexts, leading to frequent errors and less reliable translations. Similarly, Jabak (2019) concluded that GT is not a reliable tool for translating between Arabic and English. Jabak’s study, which involved an error analysis of GT outputs compared to professional translations, identified frequent lexical and syntactic errors, as well as a tendency for GT to produce literal or unintelligible translations. These findings reinforce Alsalem’s (2019) observations, highlighting that GT faces significant challenges in accurately processing complex Arabic grammar and idiomatic expressions.

Conversely, the current study examined the reverse direction: English to Arabic translation, where the input language is English. Machine translation models are primarily trained on extensive English corpora, which allows them to process English inputs with greater fluency and accuracy. Therefore, the direction of translation plays a crucial role in determining the quality of machine translation outputs. This distinction may help explain why, unlike in the studies of Alsalem (2019) and Jabak (2019), GT translations were rated higher than student translations in the current study, as GT is better equipped to process English inputs than Arabic ones.

It is also important to recognize that the challenges previously identified with Arabic-English translation may have been mitigated with the advent of newer machine translation models. A more recent study by Muftah (2022) found that machine translation systems such as GT have significantly improved, producing translations comparable to those created by human translators, even in Arabic-English contexts. Muftah’s study reported no statistically significant difference in translation adequacy between machine translation and human translations when compared to reference translations. This suggests that the quality gap between machine translation and human translation is narrowing, likely due to rapid advancements in AI technologies and neural machine translation models.

## Conclusion and recommendations

The primary goal of this study was to explore how ESL instructors rate the quality of English-to-Arabic translations produced by GT compared to those generated by student translators. The findings revealed a significant preference for GT over student translations, challenging the skepticism many educators hold regarding the reliability of machine translation tools. Contrary to the prevailing belief that machine translation systems are inherently flawed, the results demonstrate that GT can produce translations that outperform those of students nearing the completion of their translation training.

Furthermore, the study uncovered an interesting disconnect between instructors’ perceptions of machine translation quality and their actual ratings. Although machine-generated translations received significantly higher ratings overall, instructors often attributed the high-quality translations to students and the poorer ones to the GT. This preconceived notion about the quality of machine translation may reinforce educators’ reluctance to integrate machine translation into their teaching and cloud their judgment about its role in translation education. As a result, they may overlook the growing importance of machine translation tools in preparing students for the evolving demands of the translation profession. The gap between educator perceptions and machine translation’s actual performance suggests potential bias, highlighting the need for empirical research to refine these views.

There are several important implications for translation education. A key takeaway is the need to reconsider the role of machine translation tools in the classroom. Rather than enforcing an outright ban, educators should adopt a more balanced approach that leverages the potential of machine translation as a learning aid. Instructors could integrate it into translation activities that promote critical engagement. For example, students can be asked to revise and improve machine translation outputs through post-editing tasks, which help sharpen their linguistic awareness and translation judgment. This method enables students to strengthen their skills without becoming overly dependent on the technology. As several studies have shown (Correa, 2014; Kazemzadeh and Fard Kashani, 2014; Niño, 2009; Salinas and Burbat, 2023), students frequently rely on online translators even when instructed not to. This trend points to the need for a more supportive and realistic instructional environment—one that acknowledges the widespread use of machine translation tools and guides students in using them responsibly. Rather than seeking to eliminate their use, educators should focus on developing students’ competence in using machine translation effectively, including teaching post-editing strategies. By embracing the evolving role of machine translation and adapting pedagogical practices accordingly, instructors can better prepare students for a professional landscape where AI-assisted translation will likely be the norm.

## Limitations and future research

While the current study provides valuable insights into professors’ evaluations of human and machine translations, several limitations should be acknowledged. First, although the dataset comprised 968 individual ratings, those ratings were generated by only 22 instructors evaluating translations produced by 20 students. The limited number of raters and translators reduces the statistical generalizability of the findings. Future work should replicate the study with a larger and more demographically diverse pool of instructors and students to test the stability of the observed effects.

Second, the study relied on relatively short sentences extracted from student work. While machine translation systems often perform well on shorter segments, they may encounter greater challenges when processing longer texts that require maintaining coherence, consistency, and deeper contextual understanding. Future research should consider expanding the range of translation tasks to include longer and more complex texts, allowing for a broader assessment of machine translation performance across different genres and levels of difficulty.

Another limitation lies in the fact that professors were not asked to justify their ratings, which could have provided richer qualitative insights into their evaluation criteria and potential biases. Incorporating think-aloud protocols or follow-up interviews with evaluators could shed light on the cognitive processes and perceptions underlying their ratings. Finally, because the current study focused on translations between English and Arabic, the findings may not necessarily generalize to other language pairs or translation directions.

Future research should also investigate whether recent improvements in machine translation models have enhanced Arabic-to-English translation quality. While the current study found strong performance in English-to-Arabic translation, it remains unclear whether machine translation systems can similarly process Arabic input and produce high-quality English outputs.

Ethics Statement: The study used previously submitted student translations collected from past coursework. The samples were provided by colleague instructors, and all identifying information had been removed before sharing them with the researcher. The researcher did not have access to student names or contact details, and most students could not be reached. As the data functioned as an anonymized corpus, individual consent was not feasible. However, confidentiality and anonymity were maintained throughout the research.

## Data Availability

The raw data supporting the conclusions of this article will be made available by the authors, without undue reservation.
